# 

**Published:** 1874-06

**Authors:** 


### TABLE OF CONTENTS.

ORIGINAL COMMUNICATIONS.

Hypodermic Medication. By J. C. Bishop, M. D. - - 315

Tetanus and Strychnia Poisoning—their Symptoms and Differential Diagnosis. By W. P. Wells, M. D. - - - - 333

REMARKS, GLEANINGS AND EXTRACTS.

Physical Traits of Epileptics—340. Commotio Retinæ—341. Signs of Death—342. What Food is most Digestible—343. Influence of Alcohol on the Temperature of the Human Body—344. Regeneration of the Crystalline Lens in some Mammals—345. Spontaneous Retinal Pulsation as a Symptom in Basedows Disease; Remarks on Embolism—346. Physiology of the Secretion of Tears; Pathogenesis of Tumors; Food for Infants—347. Sleeping Sickaess; Indigesiion—348. Prurigo Ferox; Lotion for Fetid Feet—349. By S. M. Burnett, M. D.

Ergot in Dysentery—349. Blood-letting in Peurperal Convulsions; A Dangerous Paper—350. Podalic Version; Cinquo-Quinine; Anthemis Cotula—351. New Plan of Extracting Bodies from the Ear; A Medical Witness Answers Back; Vomiting of Pregnancy—352. By R. C. Word, M.D.

Insulation in the Treatment of Rheumatism and other Diseases—353. On the Use of the Uterine Sound in the Diagnosis of Organic Dilatation of the Uterus Predisposed to Hemorrhage—354. Formation of True Bone in Penis—355. Some Sources of Syphilitic Infection—356. A Simple Plug for Rectal Hemorrhage; Phosphoric Acid in the Urine in cases of Disease of the Brain—357. Double Spleen and Kidneys; Death from Lancing the Gum. By T. Curtis Smith, M. D.

Diphtheria—359; Cerebro-Spinal Meningitis; Action of Cold Water on the Spleen—361. Chloral Hydrate Successfully used in Tetanus; A Vegetable Cure for Syphilis—362. How to check Coughs; Whooping Cough; Treatment of Diabetes—363. Eczema Capitis; Gelseminum in Gynæcologia—364. By A. R. Kilpatrick, M. D.

Phosphastic Food in Debility—364. Cancer—365. Amyl Nitrate and its Uses—366. A Hint in giving Iodide of Potassium—367. Chloral Hydrate and Camphor as a Local Application in Neuralgia: Administration of Bromtde of Potassium—368. Treatment of Delerium Tremens; On Nervous Pharyngitis—369. Sciatica promptly cured by Croton Chloral Hydrate; New Treatment of Cancer; Chloral and Morphia in Eclaupsia; Electricity in Cerebro-Spinal Meningitis—370. Ergotin Injections in Prolaysus Ani; Pepsin; Pomade for Intertrigo and Eruptions produced by Repeated Scratching; Bromide of Potassium in Dental Irritation—371. By Local Editors.

EDITORIAL AND MISCELLANEOUS.

Manual of Eye-Surgery; Annual Report of the Supervising Surgeon of the Marine Hospital Service of the United States; Treatment of Nervous and Rheumatic Affections by Static Electricity - - - - . - - 372

Receipts of Pamphlets and Monthlies - - - - - - - - - 373

